# Associations between Level and Change in Physical Function and Brain Volumes

**DOI:** 10.1371/journal.pone.0080386

**Published:** 2013-11-12

**Authors:** Benjamin S. Aribisala, Alan J. Gow, Mark E. Bastin, Maria del Carmen Valdés Hernández, Catherine Murray, Natalie A. Royle, Susana Muñoz Maniega, John M. Starr, Ian J. Deary, Joanna M. Wardlaw

**Affiliations:** 1 Brain Research Imaging Centre, Division of Neuroimaging Sciences, School of Clinical Sciences, University of Edinburgh, Edinburgh, United Kingdom; 2 Centre for Cognitive Ageing and Cognitive Epidemiology, University of Edinburgh, Edinburgh, United Kingdom; 3 SINAPSE (Scottish Imaging Network, A Platform for Scientific Excellence) Collaboration, Edinburgh, United Kingdom; 4 Department of Computer Science, Faculty of Science, Lagos State University, Lagos, Nigeria; 5 Department of Psychology, School of Life Sciences, Heriot-Watt University, Edinburgh, United Kingdom; 6 Department of Psychology, University of Edinburgh, Edinburgh, United Kingdom; 7 Alzheimer Scotland Dementia Research Centre, University of Edinburgh, Edinburgh, United Kingdom,; University of Maryland, College Park, United States of America

## Abstract

**Background:**

Higher levels of fitness or physical function are positively associated with cognitive outcomes but the potential underlying mechanisms via brain structure are still to be elucidated in detail. We examined associations between brain structure and physical function (contemporaneous and change over the previous three years) in community-dwelling older adults.

**Methodology/Principal Findings:**

Participants from the Lothian Birth Cohort 1936 (N=694) underwent brain MRI at age 73 years to assess intracranial volume, and the volumes of total brain tissue, ventricles, grey matter, normal-appearing white matter, and white matter lesions. At ages 70 and 73, physical function was assessed by 6-meter walk, grip strength, and forced expiratory volume. A summary ‘physical function factor’ was derived from the individual measures using principal components analysis. Performance on each individual physical function measure declined across the three year interval (p<0.001). Higher level of physical function at ages 70 and 73 was associated with larger total brain tissue and white matter volumes, and smaller ventricular and white matter lesion volumes (standardized β ranged in magnitude from 0.07 to 0.17, *p*<0.001 to 0.034). Decline in physical function from age 70 to 73 was associated with smaller white matter volume (0.08, *p*<0.01, though not after correction for multiple testing), but not with any other brain volumetric measurements.

**Conclusions/Significance:**

Physical function was related to brain volumes in community-dwelling older adults: declining physical function was associated with less white matter tissue. Further study is required to explore the detailed mechanisms through which physical function might influence brain structure, and vice versa.

## Introduction

Identifying factors which reduce the rate or extent of age-related cognitive decline is a research priority [1]. Older adults who are more physically active, who take more exercise or who are fitter generally have better brain health, for example as indicated by higher levels of cognitive ability and reduced cognitive decline [2,3]. Mechanisms involving direct influences on brain structure have been proposed to explain these associations [3-8]. Although some studies have focused on identifying the links between physical activity or fitness and specific brain structures, others have demonstrated associations with global structural brain parameters and physical activity/fitness, including a positive association with grey matter volumes [5]. The current study examined the associations between objectively measured indicators of physical function (which have often been described in previous literature within the context of general bodily fitness, discussed below), and three-year change in these, and a range of volumetric structural brain parameters.

 The association between higher levels of physical activity and better cognitive function has been widely replicated [1], including in the sample used in the present study: participants reporting higher levels of physical activity at age 70 years performed better on tests of general cognitive ability and processing speed at that age [9]. The cognitively beneficial effect of increased physical activity or better fitness is so consistently reported that fitness-promoting interventions are mooted as an accessible and cost-effective method of improving, maintaining, or enhancing the cognitive health of older adults [4]. Though the phenotypic associations between physical activity or fitness and cognitive ability are well-established, there remain many unanswered questions regarding the mechanisms underlying how fitness affects cognitive ability and change [4], and specifically the causal direction of the reported associations. The mechanistic pathways might be at the molecular level, by increasing levels of factors associated with neuronal growth, or at the systems level, for example by enhancing regional brain connectivity [4]. Although some studies have focused on specific brain structures, such as the hippocampus, others have demonstrated more general whole-brain benefits of higher physical activity or better fitness. For example, a recent review noted that higher levels of physical activity were associated with greater grey matter density [5]. Fewer studies, however, have considered effects on white matter [5], though this has become an increasing focus in recent years. For example, Johnson et al. [10] reported that older adults with higher cardiorespiratory fitness had higher integrity of white matter in the corpus callosum.

 There is an extensive and growing literature on the link between physical activity and aerobic or cardiorespiratory fitness and structural brain parameters, though it will not be reviewed in further detail, given the good overviews provided by recent studies [10-12], and the conceptual review by Erickson and colleagues [4]. In addition, however, recent intervention and training studies are providing important insights into the causal nature of the reported activity/fitness associations in ever greater resolution. For example, a one-year aerobic intervention trial reported benefits of aerobic exercise both structurally, in terms of white matter integrity in the frontal and temporal lobes [13], and functionally, where those in the exercise condition had better functional connectivity [14]. Studies such as these provide support for improving fitness as a preventative strategy against cognitive decline, though given that the current study is observational in nature, these studies will not currently be considered in detail.

 Within the literature reporting the association between fitness and cognitive or brain imaging outcomes, the preferred method of assessment generally considers cardio-respiratory capacity, such as VO_2_ max, for example 5. However, studies have also included alternative, generally non-aerobic measures, and discussed the associations reported with these measures within the context of potential fitness mechanisms, an approach which the current analysis follows. These have included what might be described as more functional measures of physical capacity, performance or capability, including objective assessments of grip strength or lung capacity, for example 15, or by self-reports of functional capacity [16]. Though these markers might be considered *indicators* of physical fitness [15], they will currently be referred to as physical function to distinguish them from the gold-standard, aerobic measures [5].That is, they might describe an individual's capacity to participate in physical activity or exercise for fitness purposes, but they do not reveal actual participation or reflect fitness, per se. Limitations in utilising such measures will be considered further in the Discussion.

In the present study, the objective was to identify associations between physical function and a range of structural brain parameters, associations which might ultimately underlie the physical activity/fitness-cognitive ability associations (although the current study is not designed to make causal attributions). Previously in this sample of community-dwelling individuals in their 70s, a relatively crude measure of self-reported physical activity at age 70 was associated with less brain atrophy and reduced white matter lesion (WML) load three years later [17]. Here, we extend that analysis in two ways: by considering objectively-assessed indicators of physical function (comprising grip strength, lung function and walking speed [15]), and by considering not only the level of these physical function indicators, but also their change over time (each indicator of physical function was measured twice across a three year interval). Examining change in physical function across time is an improvement on previous studies; for example, in the related literature of 12 studies summarised in a recent review examining associations between fitness or physical activity and brain volume, only one was longitudinal [4]. Although the studies all reported that higher levels of fitness, exercise or activity were associated with larger brain volumes (for example, whole brain, cortical, white and grey matter volumes, etc.), the causal direction remained ambiguous [4]. If increasing physical activity or improving fitness are to be proposed as part of a neuroprotective strategy, then studies must demonstrate that it is improved or maintained fitness, or less decline in fitness, which predicts the structural brain parameters rather than vice versa [5]. While the current study cannot fully address the causality issue, given a longitudinal element in the assessments of physical function, it can at least examine the existence of associations between change in physical function and the structural parameters assessed.

The current analysis examined various volumetric measures of brain structure and objectively-assessed indicators of physical function, comprising grip strength, lung function and walking speed. This suite of physical function measures has previously been used by our research team to define a latent measure of physical fitness [15], though as noted above, we refer to it here as physical function to distinguish it from aerobic fitness assessments. These measures were available on two occasions, at ages 70 and 73 years. This allowed associations to be computed between *level* of physical function and volumetric brain assessments, and importantly, to determine whether *change* in physical function across three years was associated with brain volumetric measures at age 73.

## Methods

### Ethics Statement

Ethical approval for the study was obtained from the Lothian and Scotland A Research Ethics Committees, and participants gave written, informed consent.

### Participants

Participants are members of the Lothian Birth Cohort 1936 (LBC1936) [18-20]. All were born in 1936 and most took part in the Scottish Mental Survey of 1947 (SMS1947) [21]. At mean age 70 years (Wave 1), 1091 participants undertook detailed cognitive, medical and genetic testing, and provided lifestyle information [18]. Three years later (Wave 2), repeat cognitive testing was conducted (N = 866) [19] in addition to brain MRI scanning (N = 700) [20]. Mean follow-up time between Waves 1 and 2 was 3.0 years (SD = 0.28), and between the Wave 2 clinical appointment and MRI scanning was 2.2 months (SD = 1.32). 

### Brain MRI Acquisition

All brain MRI data were acquired on a GE Signa Horizon HDx 1.5 T clinical scanner (General Electric, Milwaukee, WI, USA) using a self-shielding gradient set with maximum gradient strength of 33 mT/m, and an 8-channel phased-array head coil. The imaging protocol has been described in detail elsewhere [20] and included: T_1_-, T_2_-, T_2_*-weighted and fluid-attenuated inversion recovery (FLAIR) whole brain scans.

### Brain Tissue Volume Measurements

All image analysis was performed blind to the clinical and physical fitness data. Structural scans were co-registered to the T_2_-weighted volumes using FLIRT [22] (http://www.fmrib.ox.ac.uk/fsl). A validated multispectral image processing tool, MCMxxxVI (www.sourceforge.net/projects/bric1936) [23], was used for segmentation of brain tissue volumes to measure: intracranial volume (ICV, all soft tissue structures inside the cranial cavity including brain, dura, cerebrospinal fluid (CSF) and venous sinuses); total brain tissue volume (the actual brain tissue volume without the superficial or ventricular CSF); grey matter (GM; all grey matter in cortex and subcortical regions); normal-appearing white matter (NAWM; areas of white matter not affected by white matter lesions); ventricular volume (the lateral, third and fourth ventricles combined); and WML volumes.

### Physical Function Measurements

Physical function was measured at mean ages 70 and 73 years (Waves 1 and 2) by 6-meter walk, grip strength, and forced expiratory volume. 6-meter walk was the time in seconds to walk a measured distance of 6 meters at a normal walking pace. Grip strength was measured in kg with a Jamar hand dynamometer, three times for each hand and the best of these measurements from the dominant hand was used. Forced expiratory volume from the lungs in 1 second (FEV_1_) was measured with a microspirometer, and the best of three attempts was recorded. As in previous work [15], a general physical function ‘factor’ was derived from the three fitness measures using principal components analysis (PCA), separately for age 70 and age 73, as described in the statistical analysis section. 

### Covariates

A number of covariates were included in the analysis which are known or proposed predictors of brain structure. The covariates considered for current purposes were age in days at MRI, childhood cognitive ability at age 11 (from the Moray House Test, a test of general ability completed in the SMS1947, and referred to as age-11 IQ), number of years in education, adult social class [24] and self-reported history of cardiovascular disease (including myocardial infarction, angina, heart valve problems, abnormal heart rhythm), diabetes, hypertension (medically diagnosed and/or on antihypertensive medication), and stroke from Wave 1. Smoking status at Wave 1 was classified as current, ex- or never-smoker. Participants completed the Mini-Mental State Examination (MMSE) [25]. The test is scored out of 30 and, of the 700 participants with MRI data, 6 who had scores less than 24 at Wave 1, indicative of potential cognitive impairment [26], were excluded from the analyses.

### Statistical analyses

All statistical analyses were performed using IBM SPSS version 19 (SPSS Inc., Chicago, Ill, USA). The raw physical function measures were adjusted for height and sex. The three adjusted measures correlated in magnitude from 0.23 to 0.24 (*p* < 0.001) at age 70, and 0.18 to 0.23 (*p* < 0.001) at age 73. The adjusted physical function measures at ages 70 and 73 were compared using paired t-tests. The general physical function factors were computed by PCA of the three fitness measures completed at each wave [15]. For each wave, a single factor was suggested by the Eigenvalues greater than 1 criterion, accounting for 49% and 47% of the variance respectively.

Associations between physical function and the brain volumetric measures were examined using multivariate linear regression models. Models were run using each of the volumetric measures as the dependent (outcome) variable and each of the physical function measures as the independent variable. The first models also included age and ICV; age is a major predictor of brain atrophy, and including ICV in the model allowed us to account for variation in head size. In the next model, demographic and background variables were entered: age-11 IQ, years of education, and social class. A final set of models further included self-reported medical history of cardiovascular disease, diabetes, hypertension, and stroke, and smoking status. With the stepwise addition of the covariates, attenuation of the association between physical function and the brain volumetric measures could suggest confounding by the covariates, or that they mediate the association. The models were repeated with a measure of 3-year change in physical function, expressed as a standardised residual by regressing the age 73 physical function measures on those at age 70.

## Results

The descriptives are given for the full sample at Wave 1 (N = 1079 with MMSE ≥ 24), and for the subsample in Wave 2 with MRI data (N = 694 with MMSE ≥ 24) to allow comparisons to be computed (Table 1). The baseline covariates for the full sample including the individual physical function measures were not significantly different from those of the MRI sample. Compared to participants who attended Wave 2 but refused or were unable to undertake the MRI, the MRI subsample had a higher FEV_1_ (2.33 (0.7) versus 2.18 (0.7): *t*(847) = -2.51, *p* = 0.012); there were, however, were no significant differences for grip strength (28.90 (9.4) versus 27.90 (9.6): *t*(855) = -1.22, *p* = 0.615) or 6m walk time (4.32 (1.3) versus 4.50 (1.5): *t*(852) = 1.56, *p* = 0.119). In the MRI subsample (only participants with MMSE scores of 24 or higher at Wave 1), MMSE scores declined over the 3-year follow-up: from 28.9 (SD = 1.3) at Wave 1 to 28.8 (SD = 1.4) at Wave 2 (*t*(693) = 2.21, *p* = 0.027). Performance on each of the physical function measures declined significantly between Waves 1 and 2 (Table 1), shown by lower grip strength, longer 6m walk time, and lower FEV_1_ at Wave 2 (all *p* < 0.001).

**Table 1:** Sample descriptive.

	Full Sample (N = 1079)	MRI sample (N = 694)	
	Wave 1	Wave 1	Wave 2
Age (years)	69.5 (0.8)	69.5 (0.7)	72.5 (0.7)
Sex (% male)	50.0	52.9	
MMSE (score our of 30)	28.9 (1.3)	28.9 (1.3)	28.8 (1.4)
Education (years)	10.8 (1.1)	10.8 (1.1)	
Social class	2.4 (0.9)	2.4 (0.9)	
Smoking status			
Current smoker	144 (13.3%)	74 (10.7%)	
Ex-smoker	464 (43.0%)	314 (45.2%)	
Never-smoker	471 (43.7%)	306 (44.1%)	
Grip strength (kg)	28.9 (10.2)	29.6 (10.0)	28.9 (9.4)*
6 meter walk (s)	3.9 (1.2)	3.7 (0.9)	4.3 (1.3)*
FEV_1_ (l)	2.4 (0.7)	2.4 (0.7)	2.3 (0.7)*
Intracranial volume			1453 (142)
Total brain tissue volume			1127 (107)
Ventricular volume			35 (18)
Grey matter volume			501 (71)
Normal-appearing white matter			496 (82)
White matter lesion volume			12 (13)

Note. Participants with MMSE < 24 at Wave 1 were excluded prior to analyses, giving N = 1079 at Wave 1 and 694 in the analytical sample. Social class was coded from I (professional) to V (unskilled), class III being divided into IIIN (non-manual) and IIIM (manual), respectively [24]; married women were given the higher of their own or their husband’s social class. The physical function measurements are reported before adjustment for height and sex. All MRI volumes are reported in millilitres.

* Differences compared using paired t-tests between the physical function measures at Wave 1 and 2 in the MRI sample were significant, *p* < 0.001.

**Figure 1:**
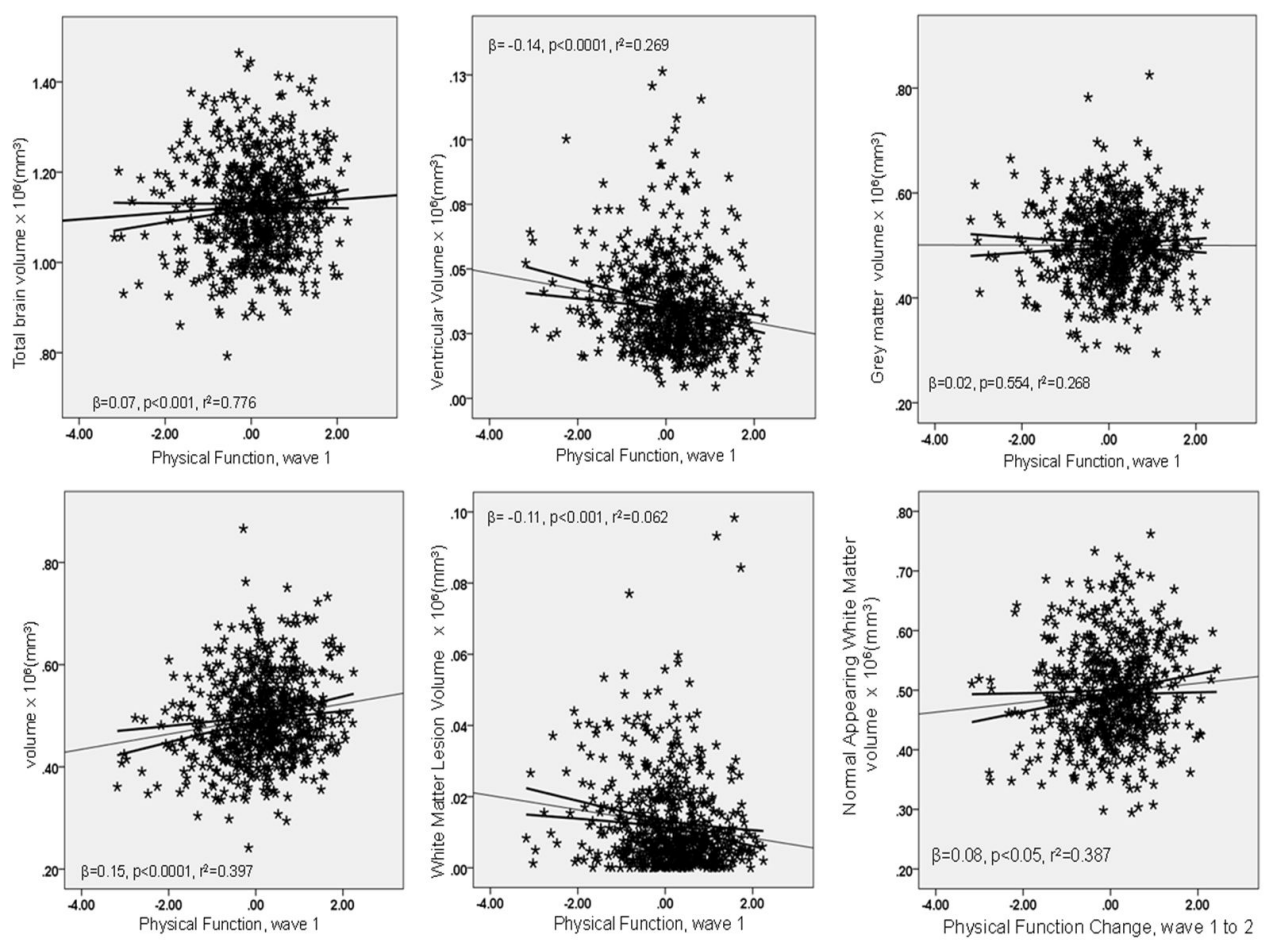
Scatterplots of physical function against brain volumetric parameters. Note. The scatterplots are from the final models accounting for all listed covariates, with the regression line and 95% C.I. displayed. The β and p-value are for the physical function measure, while the R^2^ is for the overall model. The final scatterplot shows the association between change in physical function and NAWM volume (the only significant association with change in physical function), and for clarity of illustration, extreme outliers were removed; the inclusion/exclusion of outliers did not alter the regression results. Scatterplots for the Wave 2 physical function measures were similar and are not reported here. See Figures S1-S3 for scatterplots for the individual physical function measures (extreme outliers in the final scatterplots of these figures were retained for illustrative purposes, though their inclusion/exclusion did not alter the regression results).

For brevity, only the results of the final regression analyses including all covariates are presented (Table 2 and Figure 1); the full results including details of the covariate associations with the volumetric measures are reported in Tables S1-S4. In the fully-adjusted models, a higher level of physical function at age 70 (indicated by the general physical function factor) was associated with larger total brain and NAWM volumes, and smaller WML and ventricle volumes (Table 2, and illustrated in Figure 1). The standardized β ranged in magnitude from 0.07 to 0.15 (*p* ranged from <0.001 to 0.031), with the direction of the associations reflecting whether brain tissue or lesion/ventricular volumes were being predicted. The pattern of results and the effect sizes were similar for physical function at age 73 (standardised β ranging in magnitude from 0.07 to 0.17, *p* ranged from <0.001 to 0.034). As shown in Table S1, the covariates made small additional contributions to the percentage of variance accounted for in the structural brain measures; for example, in the analyses where the R^2^ change was significant, the combination of age-11 IQ, education and social class contributed an extra 0.3% to 1.2% of accounted-for variance, whereas the health covariates only contributed to a significant change in R^2^ in the analysis of total brain tissue volume, together accounting for an additional 0.5% to 0.7% of variance.

**Table 2:** Linear regression models for the association between physical function and brain volumetric measurements.

		Total brain tissue	Ventricle	Grey matter	NAWM	WML volume
Wave 1 (age 70)	Physical function	**0.07 (<0.001**)**^+^**	**-0.14 (<0.001**)**^+^**	0.02 (0.554)	**0.15 (<0.001**)**^+^**	**-0.11 (0.009**)**^+^**
	FEV_1_	**0.05 (0.019**)**^+^**	**-0.08 (0.027**)**^+^**	-0.01 (0.826)	**0.17 (<0.001**)**^+^**	**-0.09 (0.027**)**^+^**
	Grip strength	0.03 (0.145)	**-0.10 (0.007**)**^+^**	-0.01 (0.956)	0.05 (0.153)	-0.03 (0.43)
	6m walk	**-0.07 (<0.001**)**^+^**	**0.09 (0.015**)**^+^**	-0.07 (0.069)	**-0.07 (0.031**)**^+^**	**0.11 (0.009**)**^+^**
Wave 2 (age 73)	Physical function	**0.07 (0.001**)**^+^**	**-0.12 (0.002**)**^+^**	0.02 (0.581)	**0.17 (<0.001**)**^+^**	**-0.12 (0.004**)**^+^**
	FEV_1_	0.03 (0.191)	**-0.08 (0.041)**	-0.03 (0.451)	**0.16 (<0.001**)**^+^**	**-0.09 (0.034)**
	Grip strength	0.04 (0.054)	**-0.11 (0.003**)**^+^**	0.01 (0.776)	**0.08 (0.011**)**^+^**	-0.06 (0.176)
	6m walk	**-0.07 (0.001**)**^+^**	0.05 (0.168)	-0.06 (0.081)	**-0.09 (0.008**)**^+^**	**0.11 (0.007**)**^+^**
Physical function change Wave 1 to 2	Physical function	0.02 (0.436)	-0.02 (0.636)	-0.01 (0.899)	**0.08 (0.012)**	-0.05 (0.206)
	FEV_1_	-0.02 (0.326)	-0.02 (0.602)	-0.02 (0.564)	0.01 (0.799)	-0.02 (0.560)
	Grip strength	0.02 (0.214)	-0.05 (0.177)	0.02 (0.624)	**0.07 (0.028)**	-0.05(0.252)
	6m walk	-0.03 (0.164)	-0.01 (0.932)	-0.01 (0.832)	**-0.07 (0.029)**	0.05 (0.199)

Note. Physical function factor = the general factor produced from PCA of the three measures of physical function. Values are the standardized β (and *p* value) for the listed physical function measure predicting the brain volume measures after accounting for all the covariates in the model (see Tables S1-S4 for intermediate models and the standardized β of the covariates). The models were also repeated with the physical function measures at age 73 adjusted for the relevant age 70 physical function measure as dependent variables, essentially a measure of 3-year change in physical function. The covariates included were age, ICV, age-11 IQ, years of education, social class, history of cardiovascular disease, diabetes, hypertension and stroke, and current smoking status. ^+^ represent associations that remained significant after applying a correction for false discovery rate.

The associations for the individual physical function measures (FEV_1_, grip strength and 6m walk) at ages 70 or 73 with the brain volumes were in the expected direction; namely, better physical function was associated with a greater tissue/matter volume, or smaller lesion and ventricle volume, though not all were significant (Tables S2 to S4 and Figures S1 to S3). None of the physical function variables at ages 70 or 73 were associated with grey matter. There was little attenuation on moving from baseline (age and ICV-adjusted) to the fully-adjusted models (Tables S2 to S4), suggesting that the covariates included may only partly mediate the physical function-brain volume associations.

Next, we examined how change in physical function was associated with the brain volume measurements (Table 2). The only significant associations were between change in physical function and NAWM volume, whether physical function was the general factor, grip strength or 6m walk (illustrated in the final panels of Figure 1 and Figures S2 and S3). Participants who showed least decline in physical function across three years had larger NAWM volume at age 73. The standardized β ranged in magnitude from 0.07 to 0.08 (*p* ranged from 0.012 to 0.029). The association between change in physical function and NAWM volume was not attenuated by the inclusion of the health related covariates in the models (Tables S1 to S4), again suggesting these covariates may only partly confound/mediate the reported associations.

The results reported above were not corrected for multiple testing. A correction for false-discovery rate was applied to the multiple p-values within each period (Wave 1, Wave 2, and change between Waves 1 and 2). Of the 28 significant associations, 23 remained after correction, highlighted in Table 2. In terms of the overall results with physical function for example, after applying the correction, associations remained between physical function (Waves 1 and 2) and total brain tissue volume (*p* = 0.003 and 0.005, respectively), ventricle volume (*p* = 0.003 and 0.008, respectively), NAWM (*p* = 0.003 and 0.005, respectively), and WML volume (*p* = 0.018 and 0.011, respectively). The only association with the physical function measure that did not remain was between change in physical function and NAWM (*p* = .193). Furthermore, the associations of change in grip strength and change in 6m walk with NAWM were also no longer significant after correction.

## Discussion

In this narrow-age cohort of community-dwelling older adults, physical function—defined by three objectively-measured indicators at ages 70 and 73—was associated with a range of volumetric brain parameters, including total brain tissue, ventricle, NAWM and WML volumes. Those with a better level of physical function had larger tissue volumes, smaller ventricles and less overt white matter damage. The associations reported between physical function level and NAWM or WML volume were generally the largest effect sizes observed. These associations did not diminish when covariates such as education, social class, and health status (particularly hypertension) were considered. There were fewer significant associations when change in physical function was considered, with the exception being NAWM volume: individuals showing least decline in physical function in the preceding three years had a higher NAWM volume. These associations with change did not remain after correction for multiple testing. The results are consistent with reported associations between measures of physical function or fitness and structural brain measures, and the associations with changes in physical function might be relevant to the development of interventions for cognitive decline [7,16]. Of course, the current analyses cannot address the likelihood that structural brain changes are in fact the causal factor in decline in physical function, considered below.

The main results presented are those from the final models in which a range of covariates, considered as potential confounders or mediators, were included. Comparing the initial models to the fully-adjusted models, these covariates had minimal or no attenuating effect on the associations between physical function and the brain volumetric measures. The background and demographic variables were considered as potential confounders, as previous analyses have shown that childhood cognitive ability is a determinant of brain parameters [27]. The disease history covariates were included as potential mediators, as a reduced cardiovascular risk profile associated with increased fitness might underlie the effects reported. Considering the health factors in combination, for example, added minimally to the percentage of variance accounted for in the brain volumetric measures, and only significantly so for total brain tissue volume. That these factors did not substantially account for the associations suggests, in this sample at least, that alternative mechanisms need to be empirically considered, including formal testing of mediation effects.

Consistent with cross-sectional associations from other studies [4], physical function level was associated with total brain tissue and ventricular volumes. Although fewer studies have considered white matter [5,10,13], the associations with NAWM and WML volume were generally the largest effect sizes observed. As most of the previous literature has considered grey matter, the proposed mechanistic explanations have generally addressed these associations, for example “exercise [or fitness] increases the number of new cells that are born in the hippocampus, increases the amount of capillaries, and changes the production and secretion of several neurotrophic factors and neurotransmitters” [4] (p. 40-41). In terms of the current study, this of course assumes that the aspects of physical function assessed are markers of overt fitness, which cannot be fully addressed given the lack of a measure of, for example, cardiorespiratory capacity. Studies using such physical function measures have previously invoked mechanisms via aerobic fitness measures [15], though important caveats are considered below. These physiological changes have been suggested in specific areas including the cerebellum and hippocampus [5], or resulting in increased grey matter volume more generally [7]. In the present study there were no associations with grey matter volume, either with the level of physical function or change across all measures and ages of assessment in the current analysis. Given the size of the current sample, the effect sizes are robust, with small standard errors. Previous studies have often consisted of small samples confounded by large age ranges, or have assessed grey matter density using different methods such as voxel-based approaches which may have limitations in older people [28,29]. The lack of a global association with grey matter does not, however, preclude the possibility of regionally-specific effects, and large scale replication of the current results is required. It is possible that our assessment of whole brain grey matter volume (which includes basal ganglia and cortex) may have overlooked region-specific effects. For example, Colcombe et al. [7] reported that an aerobic fitness intervention had the largest effects in the frontal lobes though they admitted their small sample limited the generalizability of the result.

 As associations between level of physical fitness or function parameters and white matter are less well-established, mechanisms to explain these are less developed [5]. Though the parameters considered currently were all structural, recent evidence from functional imaging is potentially relevant [4]. Voss et al. [30] examined the default mode network, the brain’s resting state activation, as a link between fitness and cognition, for example. Fitter individuals showed better functional connectivity, and this was suggested as a mediator of the association between aerobic fitness and executive function [30]. A more detailed examination of the aspects of white matter structure and function associated with physical fitness and function is worth pursuing.

 The strongest findings were observed with the general measure of physical function. This factor is an indicator of the shared variance of the three physical function measures used to define it, and reduces the error variance associated with any individual measure. The associations with the individual physical function measures were less consistent, though stronger effects were noted for walk time, for example. It is possible that walk time is the most sensitive measure in our battery of physical function assessments, and indeed it showed the greatest relative decline over three years; the mean change was about two thirds of a standard deviation. Methodologically, we would suggest that studies consider multiple markers of physical function or fitness and use conglomerates of these to avoid the unreliability and subsequent inconsistency of results derived from any single measure.

When change in physical function was examined, participants showing least decline in physical function had higher NAWM volume, perhaps via mechanisms similar to those above. Although physical function was examined over time, it is, however, not possible to assert physical function as a causal factor given the short time span and the absence of brain imaging data at the baseline examination. It was therefore not possible to examine the dynamic associations between change in physical function and change in the structural brain parameters. A larger decline in physical function could be an outcome of functional changes associated with declining healthy NAWM volume. Though we cannot presently distinguish between these possibilities, further physical function and MRI data being collected at age 76 will allow important cross-lagged effects to be examined. Furthermore, the associations with change did not survive correction for multiple testing; replication of these associations in other large cohorts is required.

 The more consistent effects with walk speed also suggest consideration of the current conceptual focus. As physical function measures were available on two occasions, it was the level and change in these (individually and in combination) that were generally considered as the 'predictor' variables. As noted, these were discussed within the context of fitness effects on the brain, given the large supporting literature and explanatory frameworks for those effects. Moonen and colleagues [16] reported how improvement in physical function over six years was associated with better cognitive outcomes, although they did not have brain structural measures (and their study included participants across the whole adult age range). However, and even with longitudinal physical function data, the current study cannot explore likelihood that it is the brain volumetric measures which predict changes in physical function. Studies have considered this pathway, including how age-associated changes in the brain might precede and predict changes in physical function, including assessments of gait, for example. In a recent study, loss of white matter and hippocampal atrophy were associated with a decline in step length and gait speed [31]. The study of 225 individuals had a follow-up of about 2.5 years, and was reported as the first to longitudinally consider how atrophy and prevalence of WML might influence changes in gait in older adults, though previous studies have examined aspects of the association between structural brain parameters and gait [32,33].

Associations between physical fitness, function and structural brain indices are often derived from cross-sectional studies, though there are likely to be complex interactions across time. Assessing the temporal ordering of such effects is not possible [7], though it is not uncommon to see results reported as if it were. Most studies assume a causal direction of fitness or exercise or physical activity to brain structure and cognition—for example, “exercise capacity…may be the most effective physical function in preventing structural changes of the brain associated with cognitive decline” [34]— although it is also possible that those with healthier brains are able to continue to keep their bodies healthy and in better functional condition and vice versa [33]. Studies of gait or balance parameters would usually take the opposite causal direction as the starting point: that declining structural integrity of the brain drives functional performance changes. The direction of the effects cannot be established without longitudinal follow-ups across a number of years. Such studies are particularly required if increasing physical activity or improving physical function and fitness are to be proposed as interventions to reduce cognitive decline, or indeed, that reducing age-associated changes in the brain might be an intervention strategy to improve the functional health of older adults. Longitudinal studies with broad, consistent measures of fitness, function and balance/gait across time, combined with contemporaneous assessments of brain structures, will be required to elucidate the overlapping nature of the constructs, and the causal precedence of the reported associations. Given the relative dearth of large-scale studies combining MRI assessments and long-term follow-up, studies are likely to be able to address only elements of this network of associations, as the current study aimed to do, with the proposal to continue this with repeat data collection as noted above.

### Strengths and limitations

The current study benefits from a detailed MRI protocol but, as noted, this was only completed at the age 73 assessment. Our current intention was to focus on global structural brain parameters, but it is also possible there are associations between physical function and the functional activity of the brain, including functional connectivity [30], or on volumes of specific brain regions. In terms of the latter, voxel-based approaches might be suggested, though these are not always appropriate in older samples given the greater likelihood of errors in registration to a template, hence our current focus on global parameters. Further development of the image analysis protocols in the current sample will allow regionally-specific associations to be examined in the future.

The physical function measures were objectively assessed, rather than self-reported physical activity as we and others have used previously [17], although there are other aspects of functional capacity and fitness that we did not assess. Maximal oxygen uptake is often preferred as a measure of aerobic fitness, for example. By extracting the common variance from the three measures considered to produce a general physical function variable, we attempted to address this limitation and remove error variance associated with any individual measure. Another reason for focus on global measures—both in terms of the brain imaging parameters and a latent physical function factor—was to reduce the number of analyses. Even accounting for this, the number of analyses was large, though correcting the multiple comparisons for a false-discovery rate suggested that the type I error rate was low (23 of the 28 significant associations remained). Replication of these associations in independent samples is, however, required.

Previous studies have often been conducted using small sample sizes. The samples discussed by Erickson and colleagues’ [4] review of physical activity and brain imaging parameters, for example, ranged from 52 to 299, meaning the effect sizes reported are likely to be somewhat unreliable. Though rare, larger brain imaging studies do exist: Rosano et al. [33] included almost 800 participants. The LBC1936 is therefore large with respect to previous neuroimaging studies, and the participants are well-phenotyped. As they are a year-of-birth cohort, the effect of chronological age is reduced, which would otherwise have been one of the largest and most troublesome confounders of associations between many of the variables considered. The study has a longitudinal element, albeit only three years in the present analyses. This was, however, long enough to observe declines across the physical function indicators assessed though change measures are more error prone than level measures, and both of these might reduce the likelihood of finding associations.

## Conclusions

Physical function and brain volumetric measures were significantly associated in a cohort of community-dwelling adults in their seventies, and declining physical function was associated with lower brain white matter tissue volume three years later. Though this supports improving levels of physical function, perhaps via physical fitness and activity interventions, to maintain brain health in later life, longer term follow-ups and mechanistic studies are required to explore fully the direction and nature of these associations.

## Supporting Information

Table S1 **Linear regression models for the association between physical function and brain volumetric measurements.** Note. Values are the standardized β for the listed physical function measure or covariates predicting the brain volume measures. W1 and W2 represent the physical function measurements at ages 70 and 73 years respectively, while change represents change in physical function measures. R2 is given for the overall model.
* p < 0.05, ** p < 0.01, *** p < 0.001.
(DOCX)

Table S2 **Linear regression models for the association between FEV_1_ and brain volumetric measurements.** Note. See note Table S1.
(DOCX)

Table S3 **Linear regression models for the association between grip strength and brain volumetric measurements.** Note. See note Table S1.
(DOCX)

Table S4 **Linear regression models for the association between 6m walk and brain volumetric measurements.** Note. See note Table S1.
(DOCX)

Figure S1 **Scatterplots of FEV1 against brain volumetric parameters.** Note. See note Figure 1.
(TIF)

Figure S2 **Scatterplots of grip strength against brain volumetric parameters.** Note. See note Figure 1.
(TIF)

Figure S3 **Scatterplots of grip strength against brain volumetric parameters.** Note. See note Figure 1.
(TIF)
