# Supplementary material for: Associations between Level and Change in Physical Function and Brain Volumes
Source: PLoS One. 2013 Nov 12;8(11):e80386. doi: 10.1371/journal.pone.0080386 (PMC3827194; doi:10.1371/journal.pone.0080386)
Supplement: Table S1 — Linear regression models for the association between physical function and brain volumetric measurements. Note. Values are the standardized β for the listed physical function measure or covariates predicting the brain volume measures. W1 and W2 represent the physical function measurements at ages 70 and 73 years respectively, while change represents change in physical function measures. R2 is given for the overall model. * p < 0.05, ** p < 0.01, *** p < 0.001. (DOCX) [file pone.0080386.s001.docx]

|  |  | Total Brain Tissue | | | Ventricle | | | Grey matter | | | White matter | | | WML volume | | |
| --- | --- | --- | --- | --- | --- | --- | --- | --- | --- | --- | --- | --- | --- | --- | --- | --- |
|  |  | W1 | W2 | Change | W1 | W2 | Change | W1 | W2 | Change | W1 | W2 | Change | W1 | W2 | Change |
| Model 1 | Physical function | **0.09***** | **0.09***** | 0.03 | **-0.13***** | **-0.12***** | -0.02 | 0.03 | 0.04 | 0.01 | **0.17***** | **0.18***** | **0.09^**^** | **-0.12**** | **-0.14**** | -0.07 |
|  | Age in days | **-0.08***** | **-0.07***** | **-0.09***** | 0.02 | 0.01 | 0.03 | **0.12^**^** | **0.12^**^** | **0.11**** | **-0.17***** | **-0.16***** | **-0.18***** | **0.14**** | **0.13**** | **0.14***** |
|  | ICV | **0.87***** | **0.87***** | **0.86***** | **0.48***** | **0.49***** | **0.50***** | **0.50***** | **0.50***** | **0.45***** | **0.57***** | **0.57***** | **0.56***** | 0.06 | 0.06 | 0.07 |
|  | R^2^ | .767 | .769 | .760 | .259 | .259 | .246 | .257 | .259 | .256 | .386 | .397 | .369 | .041 | .045 | .031 |
| Model 2 | Physical function | **0.09***** | **0.09***** | 0.023 | **-0.14***** | **-0.12***** | -0.02 | 0.04 | 0.04 | 0.01 | **0.16***** | **0.17***** | **0.09^**^** | **-0.13**** | **-0.14***** | -0.06 |
|  | Age in days | **-0.08***** | **-0.08***** | **-0.08***** | 0.02 | 0.01 | 0.02 | **0.12^**^** | **0.12^**^** | **0.11^**^** | **-0.17***** | **-0.15***** | **-0.17***** | **0.14**** | **0.13**** | **0.15***** |
|  | ICV | **0.87***** | **0.88***** | **0.87***** | **0.48***** | **0.48***** | **0.49***** | **0.51***** | **0.51***** | **0.50***** | **0.57***** | **0.57***** | **0.56***** | -0.08 | -0.08 | -0.08 |
|  | Age 11 IQ | 0.03 | 0.04 | 0.04 | -0.07 | -0.06 | -0.07 | 0.00 | 0.01 | 0.01 | 0.03 | 0.03 | 0.04 | -0.02 | -0.02 | -0.02 |
|  | Social class | **-0.05^*^** | -0.05 | **-0.05^**^** | 0.00 | 0.01 | 0.02 | -0.02 | 0.00 | -0.01 | **-0.09^*^** | **-0.09^*^** | **-0.11^**^** | **0.10*** | 0.11 | 0.09 |
|  | Years of Education | **-0.05***** | **-0.05***** | **-0.05** | **0.10^*^** | **0.12^*^** | **0.10^**^** | -0.04 | -0.05 | -0.04 | -0.05 | -0.05 | -0.04 | 0.05 | 0.05 | 0.06 |
|  | R^2^ | .770 | .772 | .765 | .267 | .267 | .254 | .259 | .261 | .257 | .393 | .405 | .380 | .052 | .057 | .041 |
|  | R^2^ change | **.003*** | **.004*** | **.004*** | .008 | .009 | .008 | .001 | .002 | .001 | .007 | **.009*** | **.012*** | .011 | .012 | .009 |
| Model 3 | Physical function | **0.07***** | **0.07***** | 0.02 | **-0.14***** | **-0.12^**^** | -0.02 | 0.022 | 0.02 | -0.01 | **0.15***** | **0.17***** | **0.08^*^** | **-0.11**** | **-0.12 **** | -0.05 |
|  | Age in days | **-0.08***** | **-0.08***** | **-0.09***** | 0.01 | 0.01 | 0.02 | **0.12^**^** | **0.12^**^** | **0.11^**^** | **-0.16***** | **-0.15***** | **-0.16***** | **0.13**** | **0.13**** | **0.14**** |
|  | ICV | **0.88***** | **0.88***** | **0.87***** | **0.48***** | **0.49***** | **0.49***** | **0.52***** | **0.52***** | **0.52***** | **0.57***** | **0.57***** | **0.56***** | -0.08 | -0.08 | -0.08 |
|  | Age 11 IQ | 0.02 | 0.03 | 0.03 | -0.08 | -0.07 | -0.07 | -0.01 | 0.00 | 0.00 | 0.02 | 0.03 | 0.03 | -0.03 | -0.03 | -0.03 |
|  | Social class | **-0.05^*^** | **-0.05^*^** | **-0.05^*^** | 0.00 | 0.01 | 0.01 | -0.02 | 0.00 | 0.00 | **-0.09^*^** | **-0.09^*^** | **-0.10^**^** | **0.10*** | **0.11*** | **0.10*** |
|  | Years of Education | **-0.06^*^** | **-0.06^*^** | **-0.05^*^** | **0.10^**^** | **0.10^**^** | **0.09^**^** | -0.05 | -0.05 | -0.05 | -0.06 | -0.05 | -0.05 | -0.03 | -0.03 | -0.03 |
|  | Cardiovascular | -0.04 | -0.04 | -**0.04^*^** | -0.03 | -0.03 | -0.03 | -0.041 | -0.04 | -0.04 | -0.03 | -0.02 | -0.03 | 0.03 | 0.02 | 0.04 |
|  | Diabetes | **-0.05^*^** | **-0.04** | **-0.05^*^** | 0.00 | -0.01 | 0.01 | -0.04 | -0.04 | -0.04 | -0.03 | -0.02 | -0.03 | 0.05 | 0.06 | 0.06 |
|  | Stroke | 0.01 | 0.01 | 0.01 | 0.02 | 0.02 | 0.03 | 0.00 | -0.01 | 0.00 | 0.01 | 0.02 | -0.01 | 0.06 | 0.05 | 0.06 |
|  | Smoking | **-0.04^*^** | -0.04 | **-0.05^*^** | -0.01 | -0.01 | 0.01 | **-0.07^**^** | -0.06 | **-0.07^**^** | -0.02 | -0.02 | -0.04 | 0.05 | 0.06 | 0.06 |
|  | Hypertension | 0.01 | 0.01 | 0.01 | 0.03 | 0.04 | 0.05 | 0.04 | 0.03 | 0.04 | -0.03 | -0.03 | -0.04 | 0.06 | 0.05 | 0.06 |
|  | R^2^ | .776 | .777 | .772 | .269 | .270 | .258 | .268 | .268 | .267 | .397 | .408 | .387 | .062 | .069 | .055 |
|  | R^2^ change | **.005*** | **.005*** | **.007**** | .002 | .003 | .003 | .010 | .008 | .009 | .004 | .003 | .006 | .010 | .011 | .014 |
