# Supplementary material for: Associations between Level and Change in Physical Function and Brain Volumes
Source: PLoS One. 2013 Nov 12;8(11):e80386. doi: 10.1371/journal.pone.0080386 (PMC3827194; doi:10.1371/journal.pone.0080386)
Supplement: Table S2 — Linear regression models for the association between FEV1 and brain volumetric measurements. Note. See note Table S1. (DOCX) [file pone.0080386.s002.docx]

|  |  | Total Brain Tissue | | | Ventricle | | | Grey matter | | | White matter | | | WML volume | | |
| --- | --- | --- | --- | --- | --- | --- | --- | --- | --- | --- | --- | --- | --- | --- | --- | --- |
|  |  | W1 | W2 | Change | W1 | W2 | Change | W1 | W2 | Change | W1 | W2 | Change | W1 | W2 | Change |
| Model 1 | FEV1 | **0.07***** | **0.05^*^** | -0.02 | **-0.09^*^** | **-0.08^*^** | -0.02 | 0.02 | 0.00 | -0.01 | **0.19***** | **0.17***** | 0.01 | **-0.11**** | **-0.11**** | -0.03 |
|  | Age in days | **-0.09***** | **-0.09***** | **-0.10***** | 0.02 | 0.02 | 0.03 | **0.13***** | **0.12^**^** | **0.12***** | **-0.17***** | **-0.18***** | **-0.20***** | **0.14***** | **0.14***** | **0.15***** |
|  | ICV | **0.86***** | **0.86***** | **0.86***** | **0.49***** | **0.50***** | **0.49***** | **0.50***** | **0.50***** | **0.50***** | **0.55***** | **0.55***** | **0.56***** | 0.06 | 0.06 | 0.06 |
|  | R^2^ | .764 | .762 | .759 | .247 | .248 | .241 | .258 | .258 | .257 | .396 | .392 | .362 | .038 | .038 | .027 |
| Model 2 | FEV1 | **0.06***** | **0.04^*^** | -0.02 | **-0.08^*^** | **-0.08^*^** | -0.02 | 0.02 | 0.00 | -0.01 | **0.18***** | **0.16***** | 0.01 | **-0.11**** | **-0.11**** | -0.03 |
|  | Age in days | **-0.08***** | **-0.08***** | **-0.10***** | 0.02 | 0.02 | 0.03 | **0.12***** | **0.13^*^** | **0.12***** | **-0.17***** | **-0.17***** | **-0.19***** | **0.14***** | **0.14***** | **0.15***** |
|  | ICV | **0.86***** | **0.86***** | **0.87***** | **0.49***** | **0.49***** | **0.49***** | **0.50***** | **0.51***** | **0.51***** | **0.56***** | **0.55***** | **0.56***** | **-0.09*** | **-0.10*** | **-0.09*** |
|  | Age 11 IQ | 0.04 | 0.04 | 0.04 | **-0.08^*^** | **-0.08^*^** | **-0.08^*^** | 0.00 | 0.01 | 0.01 | 0.04 | 0.04 | 0.04 | -0.02 | -0.03 | -0.02 |
|  | Social class | **-0.05^*^** | **-0.05^*^** | **-0.06^*^** | 0.01 | 0.00 | 0.01 | -0.02 | -0.01 | -0.01 | **-0.08***** | **-0.08^*^** | **-0.10^*^** | **0.10*** | **0.10*** | **0.10*** |
|  | Years of Education | **-0.05^*^** | **-0.05^*^** | **-0.05^*^** | **0.09^*^** | **0.09***** | **0.09^*^** | -0.04 | -0.05 | -0.05 | -0.04 | -0.03 | -0.04 | 0.06 | 0.06 | 0.05 |
|  | R^2^ | .768 | .766 | .764 | .256 | .257 | .250 | .260 | .259 | .259 | .403 | .400 | .373 | .050 | .051 | .039 |
|  | R^2^ change | **.004*** | **.004*** | **.005**** | .008 | .009 | .009 | .001 | .002 | .002 | .007 | **.008*** | **.011*** | .012 | **.013*** | **.013*** |
| Model 3 | FEV1 | **0.05^*^** | 0.03 | -0.02 | **-0.08^*^** | **-0.08^*^** | -0.02 | -0.01 | -0.03 | -0.02 | **0.17***** | 0.16 | 0.01 | **-0.09**** | **-0.09*** | -0.02 |
|  | Age in days | **-0.08***** | **-0.09***** | **-0.09***** | 0.02 | 0.02 | 0.02 | **0.12***** | **0.12***** | **0.12***** | **-0.16***** | **-0.16***** | -0.18 | **0.13**** | **0.14**** | **0.14***** |
|  | ICV | **0.87***** | **0.87***** | **0.87***** | **0.49***** | **0.50***** | **0.49***** | **0.52***** | **0.52***** | **0.52***** | **0.56***** | **0.56***** | **0.56***** | **-0.09*** | **-0.10*** | **-0.09*** |
|  | Age 11 IQ | 0.03 | 0.03 | 0.03 | **-0.09^*^** | **-0.09^*^** | **-0.08***** | -0.01 | 0.00 | -0.01 | 0.03 | 0.03 | 0.03 | -0.03 | -0.04 | -0.03 |
|  | Social class | **-0.05^*^** | **-0.05^*^** | **-0.06^*^** | 0.00 | 0.00 | 0.00 | -0.02 | -0.01 | -0.01 | **-0.08***** | **-0.08^*^** | **-0.10^**^** | **0.10*** | **0.10*** | **0.10*** |
|  | Years of Education | **-0.06^*^** | **-0.06^*^** | **-0.06^*^** | **0.09^*^** | **0.09^*^** | **0.09^**^** | -0.05 | -0.05 | -0.05 | -0.04 | -0.03 | -0.04 | -0.02 | -0.03 | -0.02 |
|  | Cardiovascular | **-0.04^*^** | **-0.05^*^** | **-0.05^*^** | -0.03 | -0.03 | -0.02 | -0.05 | -0.04 | -0.04 | -0.02 | -0.03 | -0.04 | 0.03 | 0.03 | 0.04 |
|  | Diabetes | **-0.05^*^** | **-0.05^*^** | **-0.05^*^** | 0 | 0.00 | 0.01 | -0.04 | -0.04 | -0.04 | -0.03 | -0.03 | -0.04 | 0.07 | 0.07 | 0.07 |
|  | Stroke | 0 | 0 | 0 .00 | 0.03 | 0.03 | 0.04 | -0.01 | -0.01 | -0.01 | 0.01 | 0.01 | 0.00 | 0.05 | 0.05 | 0.06 |
|  | Smoking | -0.04 | **-0.04^*^** | **-0.05^*^** | -0.01 | -0.01 | 0.00 | **-0.07^*^** | -0.07 | -0.07 | 0.00 | -0.01 | -0.03 | 0.05 | 0.06 | 0.06 |
|  | Hypertension | 0.01 | 0 | 0.01 | 0.04 | 0.04 | 0.04 | 0.04 | 0.03 | 0.04 | -0.04 | -0.04 | -0.04 | 0.06 | 0.06 | 0.06 |
|  | R^2^ | .773 | .772 | .772 | .259 | .260 | .253 | .269 | .268 | .268 | .407 | .404 | .381 | .062 | .063 | .056 |
|  | R^2^ change | **.006**** | **.006**** | **.008**** | .003 | .003 | .004 | .010 | .009 | .009 | .004 | .004 | .008 | .012 | .013 | .017 |
