# Supplementary material for: Associations between Level and Change in Physical Function and Brain Volumes
Source: PLoS One. 2013 Nov 12;8(11):e80386. doi: 10.1371/journal.pone.0080386 (PMC3827194; doi:10.1371/journal.pone.0080386)
Supplement: Table S3 — Linear regression models for the association between grip strength and brain volumetric measurements. Note. See note Table S1. (DOCX) [file pone.0080386.s003.docx]

|  | |  | | Total Brain Tissue | | | | | | Ventricle | | | | | | Grey matter | | | | | | White matter | | | | | | WML volume | | | | | |
| --- | --- | --- | --- | --- | --- | --- | --- | --- | --- | --- | --- | --- | --- | --- | --- | --- | --- | --- | --- | --- | --- | --- | --- | --- | --- | --- | --- | --- | --- | --- | --- | --- | --- |
|  | |  | | W1 | | W2 | | Change | | W1 | | W2 | | Change | | W1 | | W2 | | Change | | W1 | | W2 | | Change | | W1 | | W2 | | Change | |
| Model 1 | Grip strength | | 0.04 | | **0.05^*^** | | 0.03 | | **-0.10^**^** | | **-0.11^**^** | | -0.05 | | 0.00 | | 0.02 | | 0.02 | | 0.06 | | **0.09^**^** | | **0.08^*^** | | -0.04 | | -0.07 | | -0.05 | |  |
|  | Age in days | | **-0.09***** | | **-0.09***** | | **-0.09***** | | 0.02 | | 0.02 | | 0.03 | | **0.12***** | | **0.12***** | | **0.12***** | | **-0.19***** | | **-0.18***** | | **-0.19***** | | **0.15***** | | **0.14***** | | **0.15***** | |  |
|  | ICV | | **0.87***** | | **0.87***** | | **0.86***** | | **0.48***** | | **0.49***** | | **0.49***** | | **0.50***** | | **0.50***** | | **0.50***** | | **0.56***** | | **0.56***** | | **0.56***** | | 0.06 | | 0.06 | | 0.06 | |  |
|  | R^2^ | | .761 | | .762 | | .761 | | .251 | | .252 | | .244 | | .258 | | .258 | | .258 | | .365 | | .371 | | .368 | | .027 | | .030 | | .029 | |  |
| Model 2 | Grip strength | | 0.04 | | **0.05^*^** | | 0.03 | | **-0.10^**^** | | **-0.11^**^** | | -0.05 | | 0.00 | | 0.02 | | 0.02 | | 0.06 | | **0.09^**^** | | **0.08^*^** | | -0.04 | | -0.07 | | -0.05 | |  |
|  | Age in days | | **-0.09***** | | **-0.08***** | | **-0.09***** | | 0.02 | | 0.02 | | 0.03 | | **0.12***** | | **0.12***** | | **0.12***** | | **-0.18***** | | **-0.17***** | | **-0.18***** | | **0.15***** | | **0.14***** | | **0.15***** | |  |
|  | ICV | | **0.87***** | | **0.87***** | | **0.87***** | | **0.47***** | | **0.48***** | | **0.49***** | | **0.51***** | | **0.51***** | | **0.50***** | | **0.57***** | | **0.57***** | | **0.56***** | | **-0.10*** | | **-0.09*** | | **-0.09*** | |  |
|  | Age 11 IQ | | 0.04 | | 0.04 | | 0.04 | | **-0.08***** | | -0.08 | | -0.08 | | 0.01 | | 0.01 | | 0.01 | | 0.04 | | 0.04 | | 0.04 | | -0.01 | | -0.01 | | -0.01 | |  |
|  | Social class | | **-0.05^*^** | | **-0.06^*^** | | **-0.06^*^** | | 0.02 | | 0.02 | | 0.019 | | -0.02 | | -0.02 | | -0.02 | | -**0.10^**^** | | **-0.10^**^** | | **-0.10^**^** | | **0.11*** | | **0.11*** | | **0.11*** | |  |
|  | Years of Education | | **-0.05^*^** | | **-0.05^*^** | | **-0.05^*^** | | **0.10^*^** | | **0.10^*^** | | **0.10^*^** | | -0.04 | | -0.05 | | -0.05 | | -0.04 | | -0.05 | | -0.04 | | 0.05 | | 0.05 | | 0.06 | |  |
|  | R^2^ | | .765 | | .766 | | .765 | | .260 | | .262 | | .252 | | .259 | | .260 | | .260 | | .376 | | .381 | | .379 | | .039 | | .042 | | .040 | |  |
|  | R^2^ change | | **.004*** | | **.004*** | | **.004*** | | .009 | | .009 | | .009 | | .001 | | .001 | | .001 | | **.011*** | | **.011*** | | **.011*** | | .012 | | .012 | | .012 | |  |
| Model 3 | Grip strength | | 0.03 | | 0.04 | | 0.02 | | -**0.10^**^** | | **-0.11^**^** | | -0.05 | | 0.00 | | 0.01 | | 0.02 | | 0.05 | | 0.08^*^ | | **0.07***** | | -0.03 | | -0.06 | | -0.05 | |  |
|  | Age in days | | **-0.08***** | | **-0.08***** | | **-0.08***** | | 0.02 | | 0.01 | | 0.02 | | **0.12***** | | **0.12***** | | **0.12***** | | **-0.17***** | | **-0.17***** | | **-0.17***** | | **0.14**** | | **0.14**** | | **0.14**** | |  |
|  | ICV | | **0.88***** | | **0.88***** | | **0.87***** | | **0.48***** | | **0.48***** | | **0.49***** | | **0.51***** | | **0.52***** | | **0.51***** | | **0.57***** | | **0.57***** | | **0.56***** | | -0.09 | | -0.09 | | -0.09 | |  |
|  | Age 11 IQ | | 0.03 | | 0.03 | | 0.03 | | **-0.08***** | | -0.08 | | -0.08 | | -0.01 | | -0.01 | | -0.01 | | 0.03 | | 0.03 | | 0.03 | | -0.02 | | -0.02 | | -0.02 | |  |
|  | Social class | | **-0.05^*^** | | **-0.06^*^** | | **-0.05^*^** | | 0.01 | | 0.02 | | 0.01 | | -0.01 | | -0.01 | | -0.01 | | **-0.10**** | | **-0.10**** | | **-0.10***** | | **0.11*** | | **0.11*** | | **0.11*** | |  |
|  | Years of Education | | **-0.06^*^** | | **-0.06^*^** | | **-0.06^*^** | | **0.10^*^** | | **0.10^*^** | | **0.09^*^** | | -0.05 | | -0.05 | | -0.05 | | -0.05 | | -0.05 | | -0.05 | | -0.01 | | -0.02 | | -0.02 | |  |
|  | Cardiovascular | | **-0.05^*^** | | **-0.05^*^** | | **-0.05^*^** | | -0.02 | | -0.02 | | -0.02 | | -0.05 | | -0.05 | | -0.05 | | -0.03 | | -0.03 | | -0.03 | | 0.03 | | 0.03 | | 0.03 | |  |
|  | Diabetes | | **-0.05^*^** | | **-0.05^*^** | | **-0.05^*^** | | 0.00 | | 0.00 | | 0.00 | | -0.04 | | -0.04 | | -0.04 | | -0.04 | | -0.03 | | -0.04 | | 0.07 | | 0.07 | | 0.07 | |  |
|  | Stroke | | 0.00 | | 0.00 | | 0.00 | | 0.03 | | 0.03 | | 0.03 | | -0.01 | | -0.01 | | -0.01 | | 0.00 | | 0.00 | | 0.00 | | 0.07 | | 0.07 | | 0.07 | |  |
|  | Smoking | | **-0.05^*^** | | **-0.05^*^** | | **-0.05^*^** | | 0.00 | | 0.01 | | 0.01 | | -0.07 | | -0.07 | | -0.07 | | -0.03 | | -0.04 | | -0.04 | | 0.06 | | 0.06 | | 0.06 | |  |
|  | Hypertension | | 0.01 | | 0.00 | | 0.00 | | 0.03 | | 0.04 | | 0.04 | | 0.03 | | 0.03 | | 0.03 | | -0.04 | | -0.04 | | -0.04 | | 0.06 | | 0.05 | | 0.06 | |  |
|  | R^2^ | | .772 | | .773 | | .772 | | .262 | | .264 | | .255 | | .269 | | .269 | | .269 | | .383 | | .387 | | .386 | | .055 | | .057 | | .056 | |  |
|  | R^2^ change | | **.007**** | | **.007**** | | **.007**** | | .002 | | .002 | | .003 | | .009 | | .009 | | .009 | | .007 | | .006 | | .007 | | .016 | | .015 | | .016 | |  |
