# Supplementary material for: Associations between Level and Change in Physical Function and Brain Volumes
Source: PLoS One. 2013 Nov 12;8(11):e80386. doi: 10.1371/journal.pone.0080386 (PMC3827194; doi:10.1371/journal.pone.0080386)
Supplement: Table S4 — Linear regression models for the association between 6m walk and brain volumetric measurements. Note. See note Table S1. (DOCX) [file pone.0080386.s004.docx]

|  |  | Total Brain Tissue | | | Ventricle | | | Grey matter | | | White matter | | | WML volume | | |
| --- | --- | --- | --- | --- | --- | --- | --- | --- | --- | --- | --- | --- | --- | --- | --- | --- |
|  |  | W1 | W2 | Change | W1 | W2 | Change | W1 | W2 | Change | W1 | W2 | Change | W1 | W2 | Change |
| Model 1 | 6m walk | **-0.08***** | **-0.08***** | -0.04 | **0.09^*^** | 0.06 | 0.01 | -0.06 | -0.07 | -0.02 | **-0.09^**^** | **-0.11***** | **-0.08^*^** | **0.11**** | **0.12**** | 0.07 |
|  | Age in days | **-0.09***** | **-0.08***** | **-0.08***** | 0.03 | 0.02 | 0.03 | **0.12***** | **0.13***** | **0.12***** | **-0.19***** | **-0.17***** | **-0.17***** | **0.15***** | **0.13**** | **0.14**** |
|  | ICV | **0.87***** | **0.87***** | **0.86***** | **0.49***** | **0.49***** | **0.50***** | **0.50***** | **0.51***** | **0.50***** | **0.56***** | **0.57***** | **0.56***** | 0.06 | 0.06 | 0.07 |
|  | R^2^ | .765 | .767 | .761 | .251 | .249 | .248 | .261 | .263 | .258 | .368 | .375 | .368 | .038 | .041 | .032 |
| Model 2 | 6m walk | **-0.08***** | **-0.08***** | -0.04 | **0.09^*^** | 0.06 | 0.00 | -0.07 | **-0.07^*^** | -0.02 | **-0.08^*^** | **-0.10^**^** | **-0.08^*^** | **0.11**** | **0.13**** | 0.06 |
|  | Age in days | **-0.09***** | **-0.08***** | **-0.08***** | 0.03 | 0.02 | 0.03 | **0.12***** | **0.13***** | **0.12***** | **-0.18***** | **-0.17***** | **-0.16***** | **0.15***** | **0.13**** | **0.14**** |
|  | ICV | **0.87***** | **0.87***** | **0.87***** | **0.48***** | **0.48***** | **0.49***** | **0.51***** | **0.51***** | **0.51***** | **0.57***** | **0.57***** | **0.57***** | -0.08 | -0.07 | -0.08 |
|  | Age 11 IQ | 0.03 | 0.04 | 0.04 | -0.07 | -0.07 | -0.07 | 0.00 | 0.00 | 0.01 | 0.03 | 0.03 | 0.04 | -0.02 | -0.01 | -0.01 |
|  | Social class | **-0.05^*^** | **-0.05^*^** | **-0.05^*^** | 0.01 | 0.02 | 0.03 | -0.01 | -0.01 | -0.01 | **-0.09^*^** | **-0.09***** | **-0.10^*^** | **0.11*** | **0.11*** | **0.10*** |
|  | Years of Education | **-0.05^*^** | **-0.05^*^** | -0.05 | **0.10^*^** | **0.10^*^** | **0.10***** | -0.05 | -0.04 | -0.04 | -0.05 | -0.05 | -0.04 | 0.06 | 0.05 | 0.06 |
|  | R^2^ | .768 | .771 | .765 | .260 | .258 | .256 | .263 | .265 | .259 | .376 | .384 | .378 | .049 | .052 | .041 |
|  | R^2^ change | **.003*** | **.004*** | **.004*** | .008 | .009 | .008 | .002 | .002 | .001 | .008 | **.009*** | **.010*** | .011 | .011 | .009 |
| Model 3 | 6m walk | **-0.07***** | **-0.07***** | -0.03 | **0.09^*^** | 0.05 | 0.00 | -0.07 | -0.06 | -0.01 | **-0.07^*^** | **-0.09^**^** | **-0.07^*^** | **0.11**** | **0.11**** | 0.05 |
|  | Age in days | **-0.09***** | **-0.08***** | **-0.08***** | 0.03 | 0.02 | 0.03 | 0.12*** | **0.13***** | **0.12***** | **-0.17***** | **-0.16***** | **-0.16***** | **0.14***** | **0.13**** | **0.13**** |
|  | ICV | **0.88***** | **0.88***** | **0.87***** | **0.48***** | **0.49***** | **0.49***** | **0.52***** | **0.52***** | **0.52***** | **0.57***** | **0.57***** | **0.57***** | -0.08 | -0.07 | -0.07 |
|  | Age 11 IQ | 0.02 | 0.03 | 0.03 | -0.08 | -0.07 | -0.07 | -0.01 | -0.01 | 0.00 | 0.02 | 0.03 | 0.03 | -0.03 | -0.01 | -0.01 |
|  | Social class | **-0.05^*^** | **-0.05^*^** | **-0.05^*^** | 0.01 | 0.02 | 0.02 | -0.01 | -0.01 | -0.01 | **-0.09^*^** | **-0.09^*^** | **-0.10^*^** | **0.11*** | **0.11*** | **0.10*** |
|  | Years of Education | **-0.06^*^** | **-0.06^*^** | **-0.05^*^** | **0.10^*^** | **0.10***** | **0.10***** | -0.05 | -0.05 | -0.04 | -0.05 | -0.05 | -0.05 | -0.03 | -0.02 | -0.02 |
|  | Cardiovascular | -0.04 | -0.04 | -0.04 | -0.03 | -0.02 | -0.02 | -0.04 | -0.04 | -0.04 | -0.03 | -0.03 | -0.03 | 0.04 | 0.03 | 0.04 |
|  | Diabetes | **-0.05^*^** | **-0.04^*^** | **-0.05^*^** | 0.01 | 0.01 | 0.01 | -0.04 | -0.03 | -0.04 | -0.04 | -0.03 | -0.03 | 0.05 | 0.07 | 0.06 |
|  | Stroke | 0.01 | 0.00 | 0.01 | 0.02 | 0.03 | 0.03 | 0.00 | -0.01 | 0.00 | 0.00 | 0.00 | -0.01 | 0.07 | 0.06 | 0.07 |
|  | Smoking | **-0.05^*^** | **-0.04^*^** | **-0.05^*^** | 0.01 | 0.01 | 0.01 | -**0.07^*^** | -0.07 | -0.07 | -0.03 | -0.03 | -0.03 | 0.05 | 0.05 | 0.06 |
|  | Hypertension | 0.01 | 0.01 | 0.01 | 0.04 | 0.04 | 0.04 | 0.04 | 0.04 | 0.04 | -0.04 | -0.04 | -0.04 | 0.06 | 0.05 | 0.06 |
|  | R^2^ | .775 | .776 | .771 | .262 | .261 | .259 | .272 | .273 | .268 | .382 | .389 | .384 | .063 | .066 | .055 |
|  | R^2^ change | **.007**** | **.006*** | **.007**** | .002 | .003 | .003 | .009 | .008 | .009 | .007 | .006 | .006 | .013 | .014 | .014 |
